# Supplementary material for: Impact of preschool attendance, parental stress, and parental mental health on internalizing and externalizing problems during COVID-19 lockdown measures in preschool children
Source: PLoS One. 2023 Feb 13;18(2):e0281627. doi: 10.1371/journal.pone.0281627 (PMC9925084; doi:10.1371/journal.pone.0281627)
Supplement: Attachment — Submitted filename: Response to Reviewers.docx [file pone.0281627.s001.docx]

Reviewer #1:

Dear Reviewer 1,

Thank you very much for the thorough review of our manuscript titled ‘Impact of preschool attendance, parental stress, and parental mental health on internalizing and externalizing problems during COVID-19 lockdown measures in preschool children’, submitted for publication in PLOS ONE. We have carefully revised the manuscript according to your valuable comments and recommendations. We are grateful for the helpful suggestions. Please find a detailed point-by-point response below. The respective changes in the manuscript are highlighted in red to facilitate the follow-up.

Sincerely,

The authors

Introduction
Line no 2 ,“the leading cause of disability” does this mean number one cause of disability worldwide?
Thank you very much for this question. We have adjusted the formulation to ‘which constitute the number one cause of disability’ (line 2).

Lines 96 – 99; It’s not clear whether you questioned the parents once about all three time points
Thank you very much for pointing this out. We have gladly added a sentence emphasizing that T1 and T2 are assessed retrospectively (line 106-107).

It is important for an international reader to know what sort of lockdown was in place? Were the preschools completely closed? Were there day care facilities available
We are grateful for this remark and have elaborated on the restrictions. Preschools were closed completely with the exception of so called ‘emergency care’ that was reserved for healthcare workers in the first lockdown and extended to parents that had no other options of caring for their preschool children in the second. Detailed elaborations on this have been inserted in lines 80-88.


Methods
How did the participants access the online tool? Was there any kind of invitations sent for the email lists obtained through preschools? If so, what was the response rate?
Thank you for this inquiry. Participants accessed the online tool over links that were included in digital flyers or via QR codes on printed flyers. Determining the response rate is unfortunately difficult as preschools were recruited via email and telephone where preschool educators were requested to print flyers or distribute them digitally. Whether this actually took place or how many parents have seen the flyers or the emails could not be determined objectively. Several preschools refused to participate due to being overworked and having too few colleagues to compensate, thus the rate of preschools that agreed to distribute can be reported with 69 out of 120 (57.50 %) contacted preschools agreeing to participate. We have included this information in lines 128-132.

This survey is done among a non-random sample. Therefore, the external validity of the study is questionable.
Thank you very much for voicing this concern. The present study is concerned with the benefit of preschool attendance during a lockdown period among children that also attended preschool before lockdown onset. Through recruiting over preschools and educators, the sample includes all parents that were willing to participate after inquiry with only being a parent and preschool attendance as prerequisites. All local preschools were contacted without exceptions. As the comparison is meant to be between children that continuously attended and those that had to be supervised elsewhere, we believe that the sample is representative of preschool children during the pandemic. What may be considered non-random is the high level of education among parents. Preschool attendance can be costly in Germany, especially for children below the age of 3, thus, a high level of education is likely and remains representative. We have added this information to the discussion in lines 473-476.

Line 145; Why was three tools used to assess internalizing symptoms
Thank you very much for this question. Internalizing symptoms were assessed via three tools in order to validate the CMFWQ as a detailed measure of internalizing symptoms in preschool children. As the present study is not concerned with the validation, only correlations between the studies for their validity are reported. The SDQ was used in reporting the results, as it also measures externalizing. We have added this information to the manuscript in line 181-182.

The tools used to measure attachment, parental stress and parental mental health, measures the status at the point of responding and they have been validated for that purpose. Asking about these retrospectively, using tools that are not originally intended for that purpose is quite questionable
Thank you very much for pointing this out. We have included this as a limitation in lines 533-535. While the questions have been developed to be precise and clear and showed good internal consistency for all time points, they have not been validated for retrospective assessments. We have looked for possible questionnaires, however, to our knowledge no questionnaires were available that could reliably assess the variables of interest at three time points. Furthermore, the pandemic could not be predicted along with preschool closures, thus, only retrospective assessments were possible.


Results
Line 331; unless you intend to validate the CBCL, CMFWQ why are you looking at their correlations with SDQ? If you use only SDQ in the main analysis, why were these tools used?
Thank you for this remark. As mentioned above, we intended to validate the CMFWQ in a later study. Correlations between the internalizing measures were included in order to show that developments across the reported times were comparable and showed the same relationships. We have pointed this out once more in line 348-349.

Discussion
Line 496; In my opinion, this is a major flaw in the methodology.
We are grateful for the opportunity to discuss the retrospective assessment of T1 and T2 in more detail. We fully agree that retrospective questions about subjective feelings and mental health are known to be prone to recall bias (Hipp et al., 2020) as they require more cognitive load to reply (Tourangeau & Yan, 2007). Several measures were taken in the present study to avoid this; 1) measures with short questions that are validated for the purpose of inquiring about mental health were used and 2) the period questioned was short (2 weeks) and detailed (before lockdown onset). Factors like these have been shown to improve recall accuracy, even for subjective assessments regarding health (Barsky, 2022). Another aspect is that participants tend to report the past more similar to the present instead of idealizing it (Hipp et al., 2020). This suggests that the differences observed between pre-lockdown and post-lockdown values are likely underestimated instead of being overestimated. Furthermore, even when reported 10-25 years later reports of childhood problems and family environment have shown moderate agreement (Renk et al., 2004) suggesting better recall after shorter periods (Barsky, 2002). Finally, longitudinal COVID-19 studies have reported increased parental stress during the peak lockdown time which reduced but remained elevated afterwards (Adams et al., 2021). These results are in accordance with findings from retrospectively reported time points in the present study.

Although we believe that the present results provide important conclusions about the availability of preschool care, we agree that retrospective assessment remains a limitation and have extended on this in lines 516-526.

Adams, E. L., Smith, D., Caccavale, L. J., & Bean, M. K. (2021). Parents are stressed! Patterns of parent stress across COVID-19. *Frontiers in psychiatry, 12*, 626456.

Barsky, A. J. (2002). Forgetting, fabricating, and telescoping: the instability of the medical history. *Archives of Internal Medicine, 162*, 9, 981-984. https://doi.org/10.1001/archinte.162.9.981.

Hipp, L., Bünning, M., Munnes, S. & Sauermann, A. (2020). Problems and pitfalls of retrospective survey questions in COVID-19 studies. *Survey Research Methods, 14*, 2, 109-114. <https://doi.org/10.18148/srm/2020.v14i2.7741>.

Renk, K., Roberts, R., Klein, J., Rojas‐Vilches, A., & Sieger, K. (2005). Retrospective reports of college students' childhood problems. *Journal of Clinical Psychology, 61*(3), 235-250.

Tourangeau, R. & Yan, T. (2007). Sensitive questions in surveys. *Psychological Bulletin, 133*, 5, 859-883. https://doi.org/10.1037/0033-2909.133.5.859.

Reviewer #2:

Dear Reviewer 2,

Thank you very much for the thorough review of our manuscript titled ‘Impact of preschool attendance, parental stress, and parental mental health on internalizing and externalizing problems during COVID-19 lockdown measures in preschool children’, submitted for publication in PLOS ONE. We have carefully revised the manuscript according to your valuable comments and recommendations. We are grateful for the helpful suggestions. Please find a detailed point-by-point response below. The respective changes in the manuscript are highlighted in red to facilitate the follow-up.

Sincerely,

The authors

Overall, the topic is important and interesting.
I would suggest to tighten the introduction section.
Thank you very much for this suggestion. We have overworked the introduction and hope to have improved clarity.

I do not see a conclusion section. I suggest to separate conclusion from discussion.
In conclusion please provide key message found from the study and future research directions.
Thank you very much for this recommendation, which we have gladly implemented. Additionally, we have included a future research outlook at the end of the conclusion in lines 549-550.
